# A novel internal fixation technique for the treatment of olecranon avulsion fracture

**DOI:** 10.3389/fsurg.2022.1019767

**Published:** 2023-01-16

**Authors:** Hongfei Qi, Zhong Li, Teng Ma, Bing Du, Cheng Ren, Yibo Xu, Qiang Huang, Kun Zhang, Yao Lu, Ming Li

**Affiliations:** Department of Orthopaedics and Trauma, Hong Hui Hospital, Xi’an Jiaotong University College of Medicine, Xi’an, China

**Keywords:** triceps avulsion, olecranon fracture, internal fixation, T-shaped plate, fracture

## Abstract

**Objective:**

Tension band wiring and proximal ulnar plate fixation are commonly used fixation methods for olecranon fractures. However, they may not be suitable for repairing proximal olecranon avulsion fractures. In this study, we present a novel fixation technique for the treatment of proximal avulsion fractures, which is a T-shaped plate combined with a wire.

**Materials and methods:**

Between March 2016 and May 2020, surgery was performed on 16 patients with proximal olecranon avulsion fractures by using a T-shaped plate combined with a wire fixation at our hospital. The parameters followed were fracture healing time, elbow range of motion (ROM), related functional scores (the Mayo score and the DASH score), and complications related to internal fixation.

**Results:**

The average follow-up period was 17 (14–21) months and fractures had healed in all patients included in the study, with an average fracture union of 9.25 (8–12) weeks. No patient reported fixation failure, serious infection, or revision surgery. The average ROM of the elbow joint was 123° (120–135°). The Mayo score was excellent in 11 patients and good in 5. The average DASH score was 17.75 (12–24).

**Conclusion:**

Olecranon avulsion fractures were fixed with a T-shaped steel plate combined with a steel wire, which can be used for early functional exercise and for achieving good final functional results. This method can provide stable fixation, especially in elderly patients with osteoporosis.

## Introduction

Olecranon fractures account for approximately 1% of upper limb fractures and 8%–11% of elbow fractures (1). Since the triceps tendon is inserted to the olecranon, the traction of the triceps makes most olecranon fractures unsuitable for conservative treatment (2). There are different olecranon fracture classifications such as Colton, Mayo, Schatzker, AO classification, and so on. (3–5). The modified Mayo classification divides olecranon fractures into four groups: proximal avulsion fracture, simple central fracture, comminuted central fracture, and distal olecranon fracture (6). Triceps contraction may cause a proximal avulsion fracture of the olecranon, mostly occurring in elderly people with osteoporosis (7, 8). This type of injury is followed by a higher risk of fixation failure due to a small size of the fracture fragment and attached for the triceps brachii tendon. This kind of fracture can also lead to complications after olecranon fracture internal fixation (9).

Olecranon fractures are usually treated by open reduction and internal fixation by performing different surgical techniques such as tension band wiring (TBW), proximal ulnar plate (PUP) fixation, and so on. (9, 10). TBW is the most used fixation method for olecranon fractures. However, some complications can also occur after this surgery. Romero et al. have reported that the reoperation rate is 71.7% (11). Symptomatic K-wire protrusion is a common side effect after TBW surgery, sometimes followed by a local appearance of the wound and even infection (12). Considering these postoperative side effects and complications of TBW, plates are recommended for the fixation of olecranon avulsion fractures. However, for this fracture type, only one screw should be used to fix the small proximal fracture fragment (13). The fixation is considered to provide insufficient stability, and early postoperative functional exercise may lead to fixation failure. Similarly, for elderly patients with fracture and osteoporosis, early functional exercise may not be achieved due to concerns about insufficient fixation. To overcome this problem, our center has developed a T-shaped plate combined with steel wire fixation for the treatment of olecranon avulsion fractures, and we have achieved satisfactory final results. To our knowledge, this is the first time that such a technique is being employed. The following is a report of our clinical results.

## Patients and methods

The inclusion criteria were as follows: The diagnosis should be consistent with proximal ulna avulsion fracture, warranting the use of a T-shaped plate combined with steel wire fixation, age should be >18 years, and good joint function before elbow injury is necessary.

The exclusion criteria were as follows: associated fractures of other parts of the elbow (distal humerus, radial head, coronoid process), severe complications and inoperable conditions, non-displaced olecranon avulsion fracture or with displacement <2 mm at admission to hospital, time from injury to operation >3 weeks, and open fractures.

This is a retrospective study. Between 1 March 2016 and 1 May 2020, 453 patients with olecranon fractures underwent surgical treatment at our hospital. There were 42 patients with olecranon avulsion fracture, of which 16 (5 males and 11 females) with an average age of 60 (30–80) years were treated by T-shaped plates combined with wire fixation. The remaining 26 patients underwent TBW or TBW combined with steel plate surgery, and they were excluded from the study. The patients’ demographic characteristics, injury mechanism, and maximum lateral diameter of the proximal avulsion fragment of the olecranon are presented in Table 1.

**Table 1:** Demographic data and general information.

Patient	Age (year)	Sex	Injured limbs	Dominant arm	Cause of trauma	Lateral diameter of avulsed fracture fragments (mm)	Length of stay (days)
1	30	Male	Left	No	Traffic accident	7.1	5
2	57	Female	Left	No	Fall	7.6	5
3	67	Male	Right	Yes	Fall	8.1	6
4	80	Female	Right	Yes	Fall	7.4	5
5	72	Male	Left	No	Fall	7.9	5
6	66	Female	Right	Yes	Fall	7.2	8
7	53	Female	Left	No	Traffic accident	7.3	5
8	45	Female	Right	Yes	Fall	7.1	5
9	61	Female	Left	No	Traffic accident	7.7	7
10	58	Female	Right	Yes	Fall	7.4	4
11	71	Female	Right	Yes	Fall	6.9	5
12	68	Female	Right	Yes	Traffic accident	7.9	4
13	77	Female	Left	No	Fall	6.9	5
14	52	Male	Right	Yes	Fall	8.0	7
15	49	Female	Left	No	Fall	7.8	4
16	59	Female	Right	Yes	Traffic accident	7.2	5

Standard x-rays or CT were used to measure the diameter of the olecranon avulsion fracture fragment. The average lateral diameter was 7.0 (6.9–8.1) mm. All operations were performed by the same surgeon. This study was approved by the ethics committee of the Hong Hui Hospital affiliated with Xi’an Jiaotong University, and all patients signed an informed consent form.

### Surgical technique

**Figure 1:**
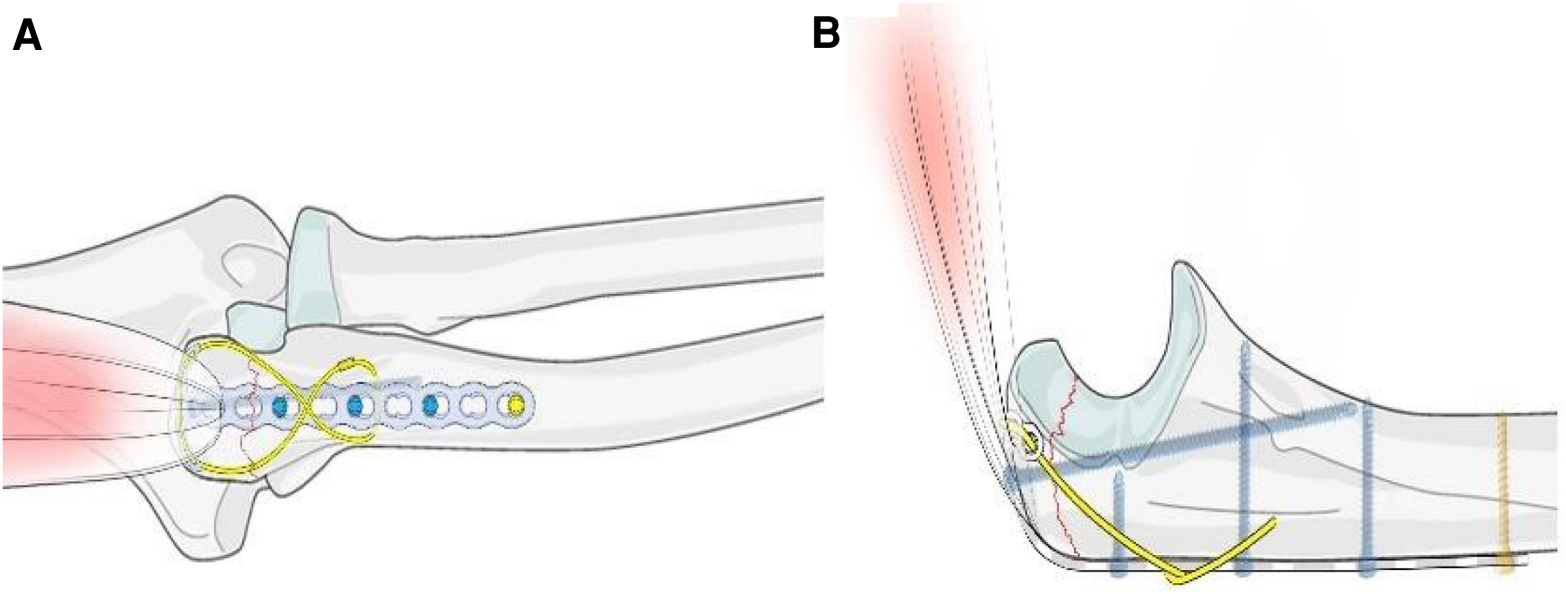
Illustration of reduction and fixation of proximal ulna avulsion fractures and elderly patients with osteoporosis using a T-plate combined with a wire. ((**A**) anteroposterior position; (**B**) lateral position).

We used a T-shaped plate and steel wire for fixation (Figures 1A,B). The transverse arm of the T-shaped plate hangs on the triceps brachii tendon to maintain reduction, and the longitudinal arm and steel wire wrap the fracture fragments in a “cage lock” way to avoid fracture displacement, share the bone surface pressure, and reduce cutting risk. This can not only offset the traction of the triceps brachii tendon but also offset the tension at the broken end of the fracture.

**Figure 2:**
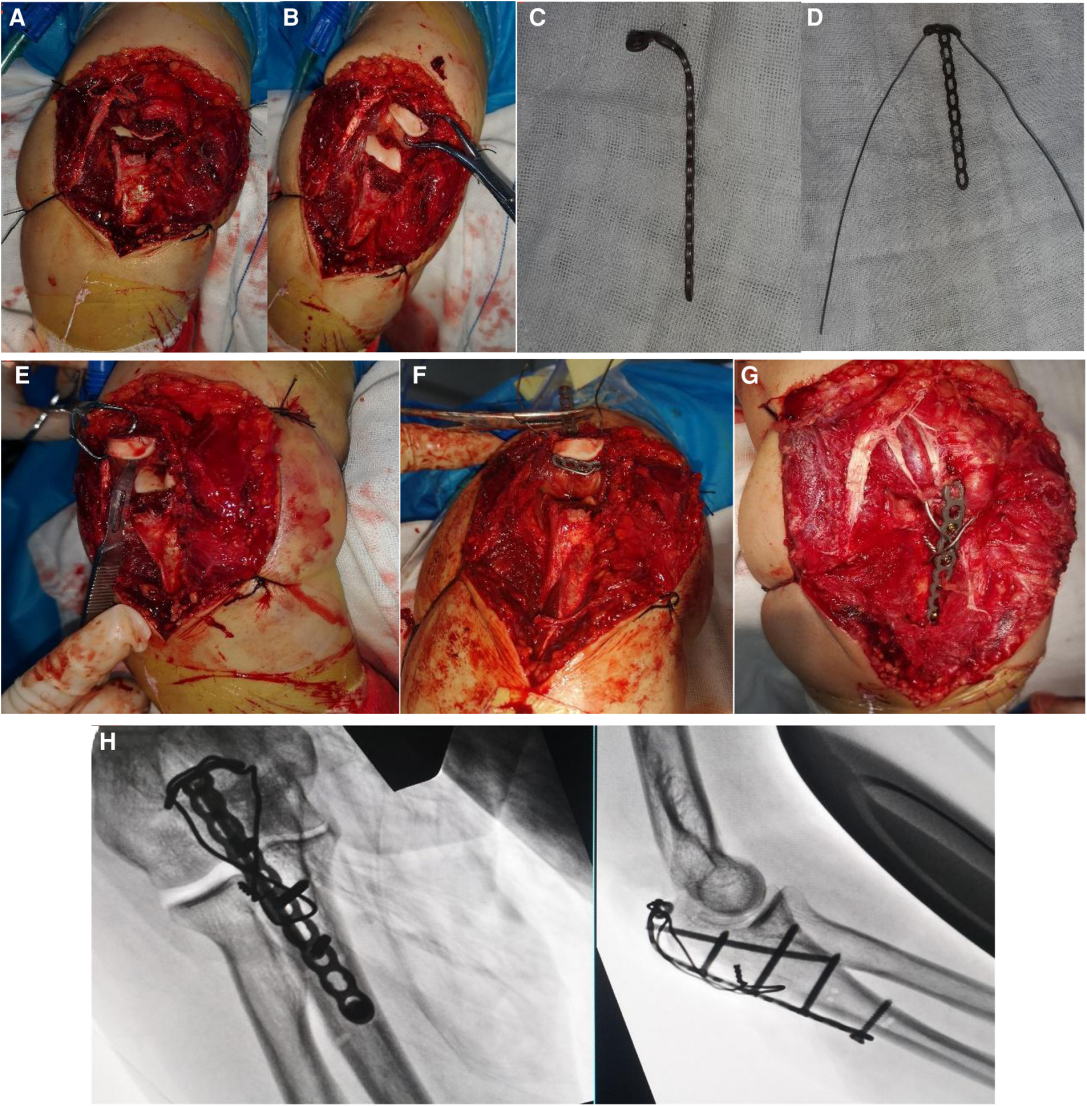
(**A,B**) Approach to the fracture; (**C,D**) bending the T-shaped plate and attaching the steel wire; (**E**) small incision in the triceps tendon; (**F**) passing the plate from the inside to the outside, thus making the T-shaped end to lean on the bare bone area of the olecranon; (**G**) pulling the triceps brachii tendon and fixing the fracture; (**H**) final operative x-ray.

A pneumatic tourniquet was used on the upper arm while the patient lay in the lateral position. Olecranon avulsion fracture was exposed after a posterior surgical approach, and joint space debridement and lavage were performed (Figures 2A,B).

After bending the T-shaped plate, a steel wire or cable was inserted through the proximal plate holes to make a loop plate (Figures 2C,D). A small incision through the triceps tendon was done by using a scalpel (Figure 2E). The loop plate was pulled in the reverse direction through the tendon incision, and the T-end of the plate was made to lean on the bony bare area of the proximal fracture fragment (Figure 2F). After fracture reduction was performed by pulling the plate distally and was maintained by employing Kirschner wires and point forceps, the screws were inserted through the plate holes. A transversal hole was drilled at the distal fracture fragment, and a wire was shaped in the form of an eight-shaped tension band (Figure 2G). The passive movements of the elbow were used to ensure normal joint movements and a stable fixation. C-arm fluoroscopy was used to check for fracture reduction and fixation quality, and then, drainage tube placement and wound closing were done (Figure 2H).

### Postoperative rehabilitation

For patients with olecranon avulsion fractures, there is no need for performing brace immobilization after surgery. The drainage tube is removed and patients are advised to start passive elbow joint movements on the second day after surgery. Active movements of the elbow joint can be started 1 week after surgery. The performance and the frequency of daily exercises are not forced on them in order to allay any apprehensions in their minds, but it is desirable that they should be performed as much as possible and as frequently as possible in the maximal range of elbow flexion and extension. Daily resistance exercises could be started 4 weeks after surgery.

### Outcome evaluation

All patients underwent an outpatient examination once a month in the first 3 months after the operation and then every 3 months. Outpatient evaluation included clinical examinations and x-ray checks. The fracture union was confirmed by x-ray presenting a continuous callus passing through the fracture line, without pain in the elbow joint during daily activities.

The range of motion (ROM) of the elbow joint was measured by placing the affected limb on a standard protractor chart. Functional results were evaluated by using the Mayo score (referred to as elbow flexion/extension range, forearm pronation/supination range, muscle strength, and pain) (14) and DASH score (referred to as upper limb diseases and upper limb function) (15). Both scores have a value range of 0–100. A higher Mayo score means a better result (95–100 is excellent, 80–94 is good, 60–79 is fair, and 0–50 is poor), while a higher DASH score means a poor result.

Complications such as fixation failure and infection and the need for revision surgery were also evaluated.

## Results

**Figure 3:**
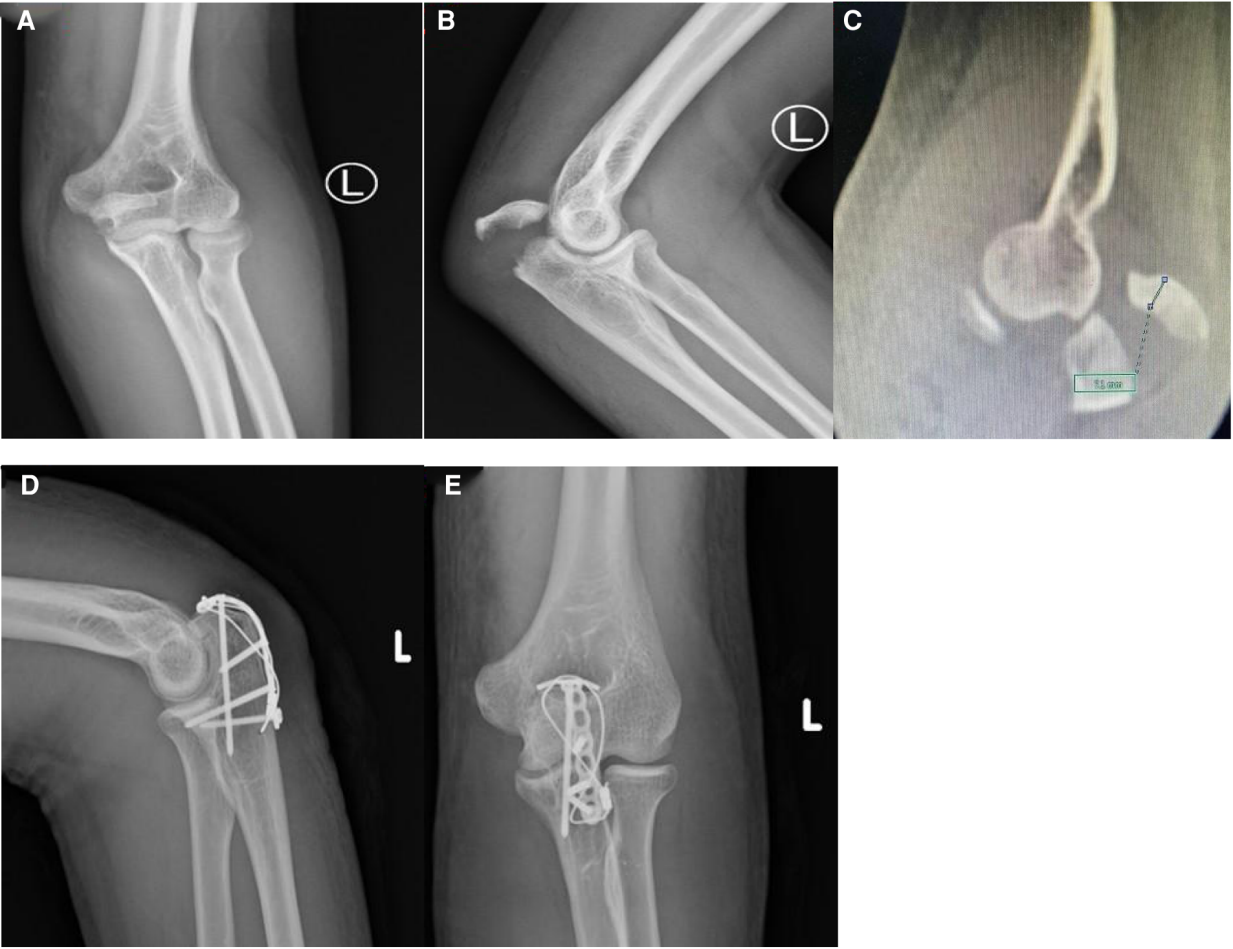
A 30-year-old male with olecranon avulsion caused by a traffic accident, treated by a T-plate combined with wire fixation; final elbow functional results were good; x-ray and CT of the elbow before surgery (**A–C**); x-ray of elbow after surgery (**D,E**).

The average follow-up time was 17 (14–21) months (Table 2). All patients had their fractures healed, and the average fracture healing time was 9.25 (8–12) weeks. No patient reported any serious complication such as fixation failure or infection and none underwent revision surgery. The average ROM of the elbow joint was 123° (120°–135°). At the last follow-up, among the 16 patients, the Mayo score was excellent in 11 patients and good in 5. The average DASH score was 17.75 (12–24). A typical case is presented in Figure 3.

**Table 2:** Clinical results and follow-up data.

Patient	Follow-up times (month)	Union times (week)	Final ROM	Mayo score	DASH score
1	18	8	120°	Excellent	12
2	16	10	120°	Excellent	16
3	14	8	125°	Good	22
4	18	8	125°	Good	20
5	17	8	125°	Excellent	18
6	19	12	120°	Excellent	18
7	16	10	125°	Good	20
8	20	8	120°	Excellent	16
9	16	12	120°	Excellent	18
10	18	8	125°	Excellent	22
11	16	8	130°	Good	16
12	14	10	125°	Excellent	14
13	18	10	130°	Excellent	18
14	17	12	120°	Excellent	14
15	21	8	120°	Good	16
16	14	8	125°	Excellent	24

## Discussion

Olecranon fractures have a bimodal distribution, as they are more common in young people with high-energy injuries and in elderly people with osteoporosis after a simple fall. Olecranon avulsion fractures are mostly found in elderly patients with osteoporosis after a simple fall (16). An epidemiological study involving 2,463 people showed that proximal avulsion fractures accounted for approximately 12% of all patients with olecranon fractures (6). Fixation of olecranon avulsion fractures has an increased rate of failure in comminuted osteoporotic bone (8, 17). Tension band wiring fixation and intramedullary screw fixation may not be suitable for correcting the osteoporotic bone, and the standard plate fixation method may also not be a stable and satisfactory one, and thus, fragment excision with triceps advancement may be required (18–20). In our study, a T-shaped plate, combined with a wire fixation technique, was used to treat olecranon avulsion fractures, which yielded satisfactory clinical results and elbow function. More detailed introduction can be found in the supplementary materials. We suggest that this technique not only fixes fracture but also offsets the tension of the triceps tendon sufficiently to provide a good strategy for further olecranon avulsion fracture treatment.

Orbay et al. (13) proposed an augmented suture of the triceps tendon directly to the plate. They found that olecranon avulsion fracture became a postoperative complication after plate fixation of an olecranon fracture, which inspired their solution. Experimental failure force of the triceps traction increased from 967.7 to 1,204.3 N. However, this suture technique was not widely used, possibly because clinicians did not pay enough attention to the postoperative complication.

TBW is the most used internal fixation method for olecranon fractures (21). A simple operating technique and low cost are the advantages of this method (22). Skin and soft tissue irritation and K-wiring protrusion are the most common postoperative complications of TBW, and many patients need a second operation to remove the internal fixation after the fracture has healed (22). TBW was thought to convert the tensile forces applied across the fracture by the longitudinal pull of the extensor mechanism to a compressive force at the fracture; however, this has not been proven either in a laboratory cyclic loading study (23) or in another biomechanical study (7). A biomechanical study reported by Wilson et al. showed that the average compressive force on the olecranon fracture surface was 77 N in TBW and 819 N in the plate fixation method (7). In addition, the characteristics of olecranon avulsion fractures include the following: small fractured fragments and the affected population comprising mostly elderly patients with osteoporosis; thus, the risk of K-wire loosening and cutting is greatly increased in TBW fixation (24).

Due to the small size of the olecranon avulsion fracture fragment, only the proximal screw can truly resist the tension of the triceps (8). To overcome this disadvantage, Wild et al. used a modified Krackow suture augmented to olecranon avulsion locking plate fixation, which resulted in a 48% increase in the median load of fixation failure (25). Izzi and Athwal used an off-loading augmented triceps suture technique in the plate fixation of comminuted osteoporotic olecranon fractures and achieved good clinical results (8). A variety of augmented suture techniques can increase the strength of PUP fixation. However, clinicians may still be concerned about early postoperative functional exercises. The reason why we did not carry out a comparative evaluation of TBW or PUP in our study is that a simple tension band or proximal plate fixation may not be suitable for repairing the proximal avulsion fracture of olecranon, for which the reinforcement repair of the triceps tendon is required.

At our center, a T-shaped plate, combined with the steel wire fixation method, is used to treat olecranon avulsion fractures. Traditionally, the plate is placed not directly on the dorsal side of the olecranon but through a small incision of the triceps tendon, and the T-shaped end of the plate is placed on the bare bony area at the proximal end of the olecranon. This technique is not about just fixing a fracture but involves maintaining the continuity of the triceps tendon and providing effective resistance to the traction of the triceps muscle. Traditional placement of the plate cannot provide sufficient adhering to the avulsion fracture fragment, and thus, the blocking effect on the tension side of the fracture is weak. The fixation method presented in this study provides for the plate to adhere to the surface of the avulsion fracture fragment more closely, and thus, the tension side fixation blocking effect is strong. Also, to provide a more stable fixation to enable one to withstand the process of physical therapy after surgery, we combined additional wiring in the form of the number “8”. We suggest that the key to the fixation of olecranon proximal avulsion fracture is how to offset the tension of the triceps brachii tendon on the fracture block. This type of fixation solves this problem well. Through the whole set of the fixation device, the continuity of mechanical conduction is restored, the fixation strength increases and becomes more reliable, and the early functional exercise of patients is promoted. The key to this technology is the plasticity of the T-shaped plate. The T-shaped end of the plate is placed in the bony bare area near the olecranon. The width of the T-shaped end should not exceed the bony bare area in order to prevent the plate from hitting the edge of the olecranon fossa. In addition, this technology requires a certain learning curve. In the initial application, it may be difficult to implement the plastic process of the plate, and thus, a long operation time may be required. Of course, the process itself will not be long, because the technology is easy to master.

The advantages of olecranon fracture surgical treatment include stable anatomical reduction, bone healing promotion, and early elbow joint activity (26). Our unique plate-wiring technique can meet the above requirements. The 16 patients included in the study had their fractures fully healed, as revealed by clear x-rays, and achieved good elbow function. No fixation failure, infection, or other complications were reported. However, in the case of one patient, indomethacin use after the operation led to heterotopic ossification, but he did not show any obvious limitation of elbow function, which may be related to the size and severity of the injury. All patients could perform active elbow functional exercises 1 week after the surgery, and exercises with resistance or gravity could be performed 4 weeks after. At the last follow-up, no patient reported soft tissue irritation around the implant.

The main limitation of our study was that it was a single-center retrospective design with a small sample size and without a control group. Also, a biomechanical analysis was not performed. Although we presented satisfactory long-term clinical results, a biomechanical research is still desirable to test the assumptions about biomechanical principles in the fixation method used. Finally, there was a limitation in that our follow-up time may not have been long enough, because a longer follow-up may lead to elaborate and different conclusions.

## Conclusions

A T-shaped plate, combined with wire fixation, for the treatment of olecranon avulsion fractures is a simple performance method, providing reliable fixation stability, early elbow flexion and extension activities, and good clinical results.

## Data Availability

The raw data supporting the conclusions of this article will be made available by the authors, without undue reservation.
