# Supplementary material for: A novel internal fixation technique for the treatment of olecranon avulsion fracture
Source: Front Surg. 2023 Jan 16;9:1019767. doi: 10.3389/fsurg.2022.1019767 (PMC9886313; doi:10.3389/fsurg.2022.1019767)

**The following is our supplementary introduction to this study:**

The first is about the concept of proximal olecranon avulsion fracture. An epidemiological study of 2462 patients in Sweden divided the olecranon fracture into the following four types according to the modified Mayo classification.
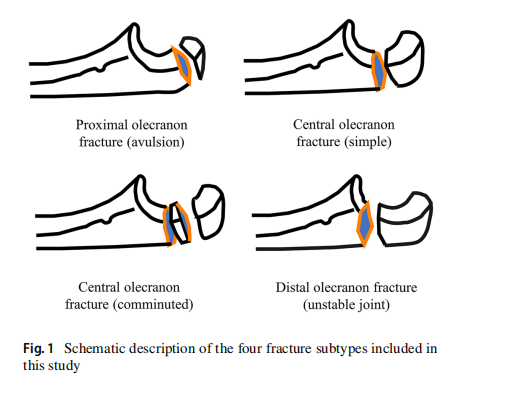


Then we think about what kind of fixation technology we choose for this kind of fracture. Because the olecranon avulsion fracture block is small, the traditional tension band and proximal ulna plate fixation cannot meet the fixation strength, the triceps brachii traction may lead to the failure of internal fixation, and the long-term brace braking after operation may affect the function of elbow joint. Our center innovatively uses T-shaped steel plate and steel wire for fixation, which can offset the traction of triceps brachii, and the fixation strength is reliable, which is conducive to the recovery of elbow function.


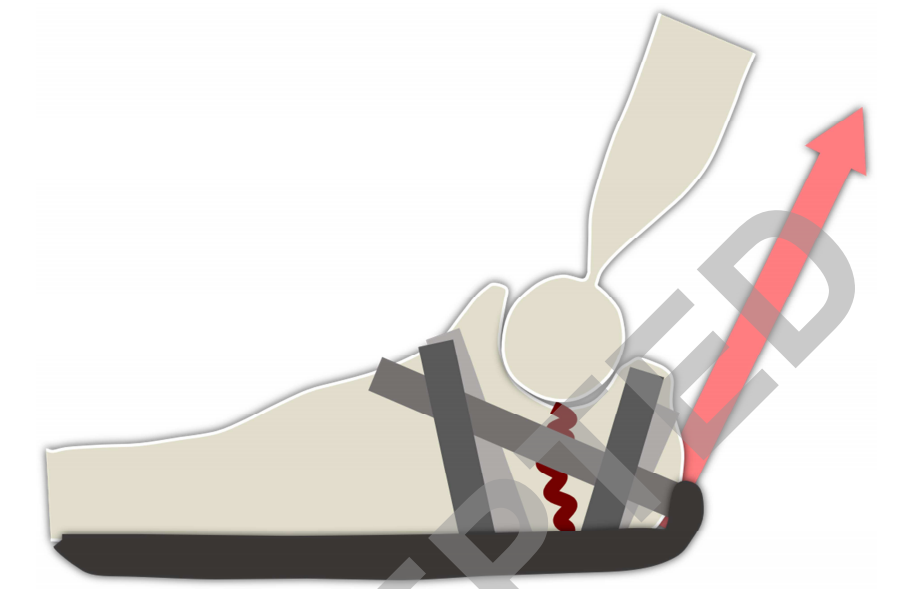


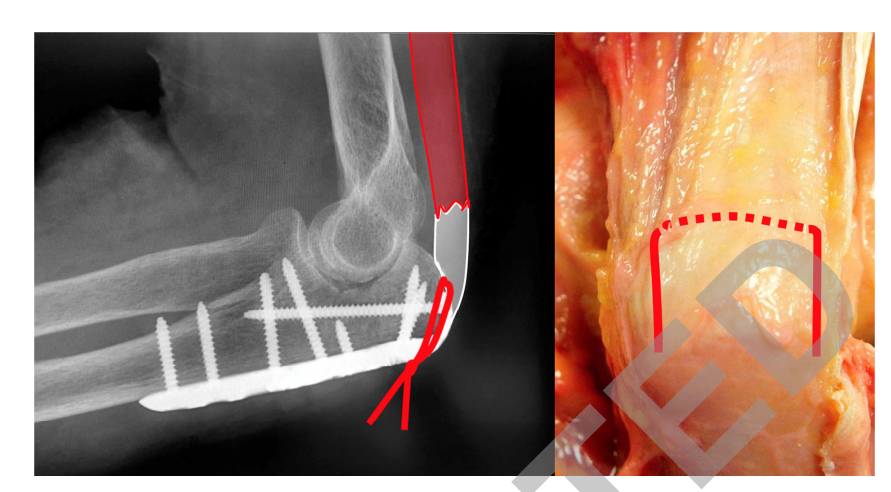


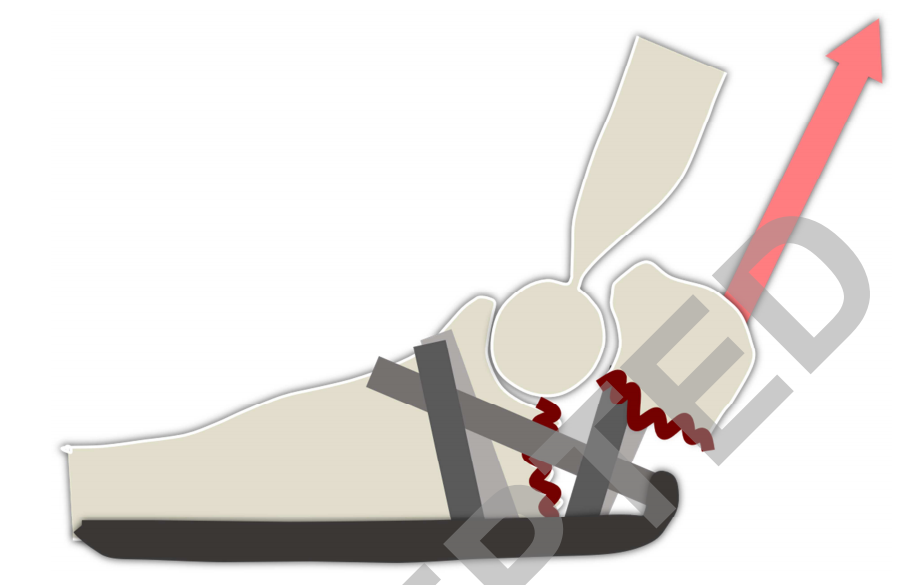


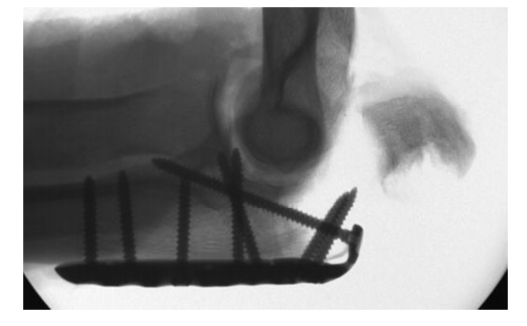


Our fixation technology can not only provide strong fixation for proximal ulna avulsion fracture, but also be conducive to early functional exercise and better elbow function. Moreover, it also has certain advantages for comminuted olecranon fractures in elderly patients with osteoporosis. T-shaped plate combined with steel wire can wrap and fix the fracture block in three dimensions.


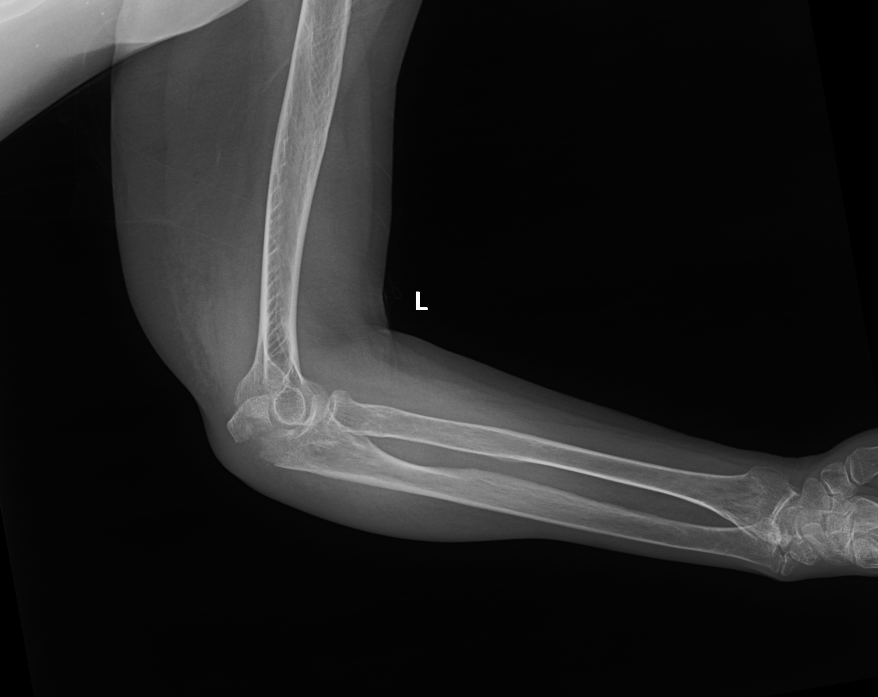

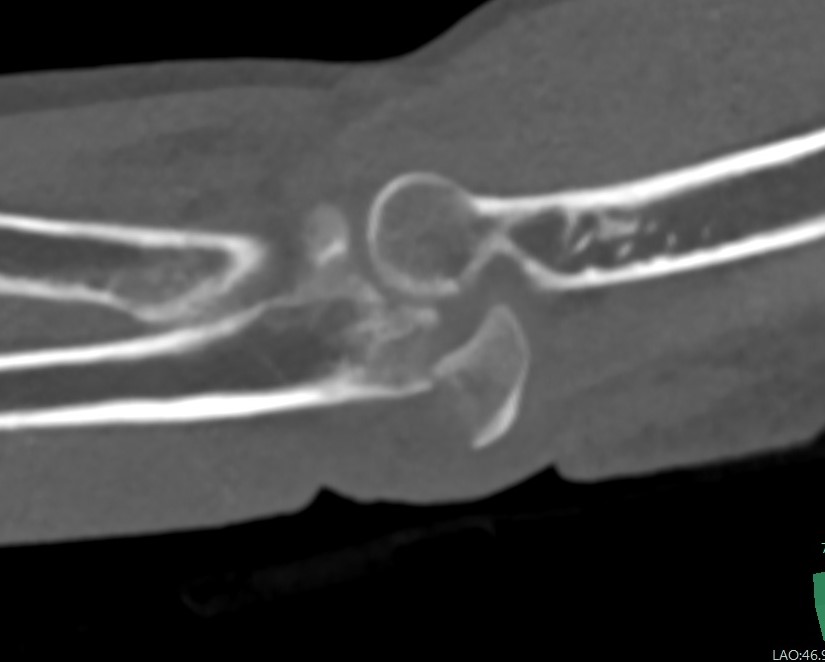


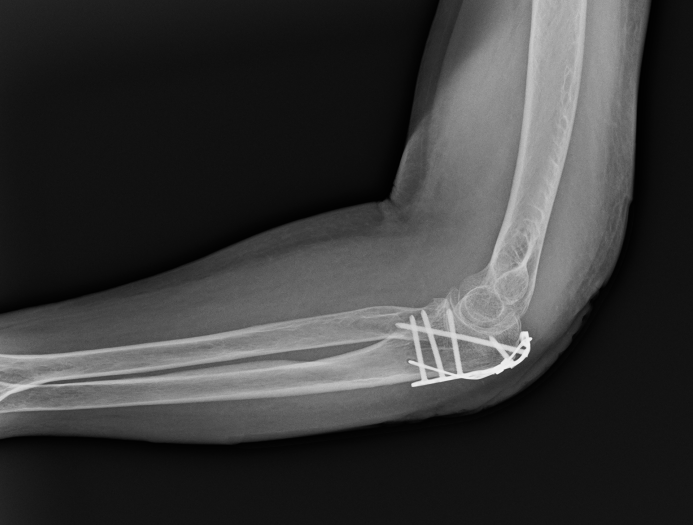

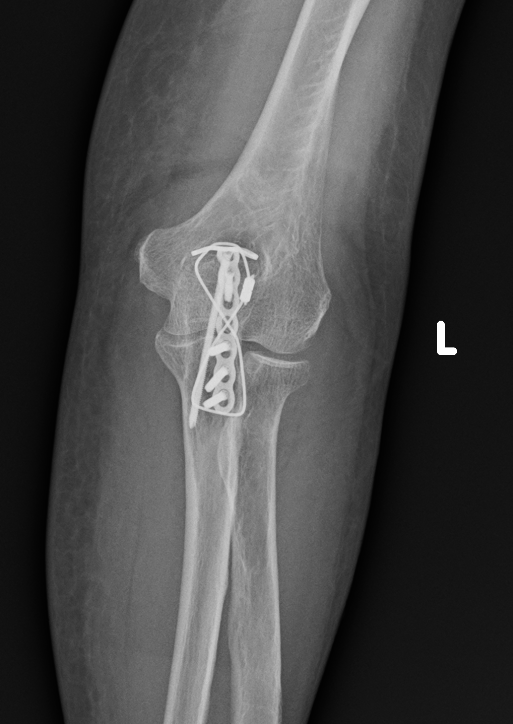

Supplement: Supplementary file 1 [file Datasheet1.docx]
